# Screening for Antibacterial and Antioxidant Activities and Phytochemical Analysis of *Oroxylum indicum* Fruit Extracts

**DOI:** 10.3390/molecules21040446

**Published:** 2016-04-07

**Authors:** Patchima Sithisarn, Petcharat Nantateerapong, Piyanuch Rojsanga, Pongtip Sithisarn

**Affiliations:** 1 Department of Veterinary Public Health, Faculty of Veterinary Medicine, Kasetsart University, Kampangsaen campus, Nakhon Pathom 73140, Thailand; fvetphs@ku.ac.th; 2 Department of Pharmacognosy, Faculty of Pharmacy, Mahidol University, Bangkok 10400, Thailand; petcharat.nan1991@hotmail.com; 3 Department of Pharmaceutical Chemistry, Faculty of Pharmacy, Mahidol University, Bangkok 10400, Thailand; piyanuch.roj@mahidol.ac.th

**Keywords:** antibacterial, antioxidant, *Oroxylum indicum*, *Streptococcus suis*, *Staphylococcus intermedius*, DPPH, Disc diffusion, TLC, total phenolic, total flavonoid, baicalein

## Abstract

*Oroxylum indicum*, which is called Pheka in Thai, is a traditional Thai plant in the Bignoniaceae family with various ethnomedical uses such as as an astringent, an anti-inflammatory agent, an anti-bronchitic agent, an anti-helminthic agent and an anti-microbial agent. The young fruits of this plant have also been consumed as vegetables. However, there has been no report concerning its antibacterial activities, especially activities related to clinically isolated pathogenic bacteria and the *in vitro* antioxidant effects of this plant. Therefore, the extracts from *O. indicum* fruits and seeds collected from different provinces in Thailand were prepared by decoction and maceration with ethanol and determined for their *in vitro* antibacterial effects on two clinically isolated bacteria, *Streptococcus suis* and *Staphylococcus intermedius*, using disc diffusion assay. Ethanol extracts from *O. indicum* fruits collected from Nakorn Pathom province at the concentration of 1000 mg/mL exhibited intermediate antibacterial activity against *S. intermedius* with an inhibition zone of 15.11 mm. Moreover, it promoted moderate inhibitory effects on *S. suis* with an inhibition zone of 14.39 mm. The extracts prepared by maceration with ethanol promoted higher antibacterial activities than those prepared with water. The ethanol extract from the seeds of this plant, purchased in Bangkok, showed stronger *in vitro* antioxidant activities than the other extracts, with an EC_50_ value of 26.33 µg/mL. Phytochemical analysis suggested that the seed ethanol extract contained the highest total phenolic and flavonoid contents (10.66 g% gallic acid equivalent and 7.16 g% quercetin equivalent, respectively) by a significant amount. Thin layer chromatographic analysis of the extracts showed the chromatographic band that could correspond to a flavonoid baicalein. From the results, extracts from *O. indicum* fruits have an *in vitro* antioxidant effect, with antibacterial potential, on clinically pathologic bacteria and they contain an antioxidant flavonoid which could be developed for medicinal and pharmaceutical purposes in the future.

## 1. Introduction

Ethnoveterinary medicine (EVM) is the traditional practice of using natural products, mainly plant extracts, to protect, treat or support animal health [1]. Natural products are sources of new chemical diversities and also pharmaceutical components. They are future antimicrobial candidates, which could provide more effective and less toxic antimicrobial compounds [2]. *Oroxylum indicum* (L.) Vent. is a medium-size, deciduous tree with various ethnomedical uses. Mature fruit is acrid and sweet, which promotes anti-helminthic and -stomachic effects [3]. The seeds have been used as purgative while the seed paste is applied to the throat for quick relief of tonsil pain [4,5]. Many flavonoids such as baicalein and biochanin A were previously reported in the pods, seeds and root bark of this plant [6,7,8,9,10,11]. Therefore, the extracts from *O. indicum* fruits and seeds collected from different provinces in Thailand were prepared by decoction and maceration with ethanol and tested for *in vitro* antibacterial effects on two clinically isolated bacteria, namely *Streptococcus suis* and *Staphylococcus intermedius*, by disc diffusion assay, and they were tested for *in vitro* antioxidant effects using the DPPH scavenging method.

## 2. Results and Discussion

### 2.1. In Vitro Antibacterial Activity of *O. indicum Extracts* Using a Disc Diffusion Assay

The antimicrobial activity of the extracts from the fruits and seeds of *O. indicum* was studied against two clinically pathogenic bacterial strains (*Staphylococcus intermedius* and *Streptococcus suis*) using disc diffusion assay. As shown in Table 1, the fruit extracts of *O. indicum* prepared by both decoction and maceration methods promoted intermediate inhibiting effects on *S. intermedius* with the inhibition zone ranging from 11–15 mm at a concentration of 1000 mg/mL. These extracts also promoted moderate inhibiting activity on *S. suis* with the zone of inhibition ranging from 10–14 mm at a concentration of 1000 mg/mL. The antibacterial activities of the extracts increased, in a dose-dependent manner, in relation to the concentration of the extracts. The dose effectiveness was compared between water and ethanol extracts, with the water extracts exhibiting less effectiveness than the ethanol extracts ranging from two-fold to 64-fold (100 to 250 mg/mL fruit extracts for *Staphylococcus intermedius*, 6.25 to 25 mg/mL seed extracts for *Staphylococcus intermedius*, 250 to 500 mg/mL fruit extracts for *Streptococcus suis*, and 1.5625 to 100 mg/mL seed extracts for *Streptococcus suis*). Correspondingly, when the dose effectiveness was compared between extracts (fruits and seeds) of two different origins , the fruit extracts delivered less inhibitory activity than the seed extracts by four-fold to 160-fold (6.25 to 100 mg/mL in *Staphylococcus intermedius* and 1.5625 to 250 mg/mL in *Streptococcus suis*). However, the seed water and ethanol extracts of this plant promoted low inhibitory effects on both bacteria.

### 2.2. In Vitro Antioxidant Activity of *O. indicum* *Extracts* by DPPH Scavenging Method

As shown in Table 2, the ethanol and water extracts from *O. indicum* seeds purchased from Bangkok (OSBE = ethanol extract from *O. indicum* seeds and OSBD = water extract from *O. indicum* seeds) significantly exhibited high antioxidant activities as determined by a DPPH scavenging assay, with EC_50_ values of 26.33 and 38.87 µg/mL, respectively. However, the water and ethanol extracts from the fruits of *O. indicum* promoted lower antioxidant effects, with EC_50_ values ranging from 65.89–220.50 µg/mL.

### 2.3. Determination Oftotal Phenolic Content in O. indicum Extracts Using the Folin-Ciocalteu Method

As shown in Table 2, the *O. indicum* extracts contained total phenolic contents in the range of 2.70 to 10.66 g gallic acid equivalent in each 100 g extract. The ethanol extract of the seeds purchased from Bangkok (OSBE) significantly contained the highest total phenolic content.

### 2.4. Determination of Total Flavonoid Content in O. indicum Extracts Using the Aluminium Chloride Method

Total flavonoid contents in *O. indicum* fruit extracts ranged from 1.49 to 7.16 g quercetin equivalent in each 100 g extract. OSBE also significantly contained the highest total flavonoid content (Table 2).

It was found that the mature seeds contained both a higher total phenolic content and a higher flavonoid content. The amounts of phenolic and flavonoid compounds showed high correlations with the antioxidant activities tested using DPPH scavenging assay (R = 0.8190 and 0.8817, respectively). These correlations could be the results of the abilities of the phenolic and flavonoid compounds in the extracts to donate protons to the DPPH free radicals [12]. There are some reports that suggest that the extraction methods have significant effects on both phenolic compounds and antioxidant activity [13,14]. From the results, preparation by maceration with ethanol promoted higher total phenolic and total flavonoid extract contents than preparation by decoction. Samatha *et al.* [15] reported that among various parts of *O. indicum* collected in India, the bark methanolic extract contained the highest total phenolic and total flavonoid contents. Reports from Moirangthem *et al.* [16] mentioned that methanol extracts from the stem bark of *O. indicum* also contained the highest total phenolic and total flavonoid contents (320.7 and 346.6 mg/g, respectively).

### 2.5. Thin Layer Chromatography (TLC) Analysis

**Figure 1:**
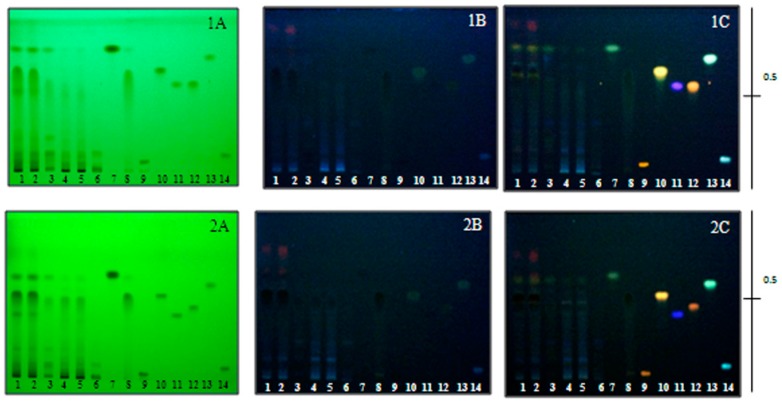
TLC chromatogram of *O. indicum* extracts; 1 = OPCE, 2 = OPCD, 3 = OPNE, 4 = OPND, 5 = OSBE, 6 = OSBD, 7 = biochanin A, 8 = baicalein, 9 = rutin, 10 = quercetin, 11 = gallic acid, 12 = myricetin, 13 = kampferol, 14 = chlorogenic acid. Adsorbent: Silica gel GF254. Solvent system: 1 = ethyl acetate-glacial acetic acid–formic acid–hexane (5:1:1:5), 2 = ethyl acetate–toluene–formic acid (25:25:7.5). Detection: A = UV 254 nm, B = UV 366 nm, C = NP/PEG spray reagent detected under UV 366 nm. 1A = ethyl acetate–glacial acetic acid–formic acid–hexane (5:1:1:5) as solvent system, detect under UV 254 nm, 1B = ethyl acetate–glacial acetic acid–formic acid–hexane (5:1:1:5) as solvent system, detect under in UV 366 nm, 1C = ethyl acetate–glacial acetic acid–formic acid–hexane (5:1:1:5) as solvent system, detect after spray with NP/PEG spray reagent under UV 366 nm, 2A = ethyl acetate–toluene–formic acid (25:25:7.5) as solvent system, detect under in UV 254 nm, 2B = ethyl acetate–toluene–formic acid (25:25:7.5) as solvent system, detect under in UV 366 nm, 2C = ethyl acetate–toluene–formic acid (25:25:7.5) as solvent system, detect after spray with NP/PEG spray reagent under UV 366 nm.

All *O. indicum* extracts were phytochemically analyzed using two different solvent systems. They exhibited TLC fingerprints as shown in Figure 1, with the presence of chromatographic bands that corresponded to some phenolics and flavonoids. In solvent system 1, there was a chromatographic band that could correspond to baicalein at an R*_f_* value of 0.65 while in solvent system 2, it was detected at an R*_f_* value of 0.53. This compound exhibited a DPPH scavenging effect with an EC_50_ value of 3.17 µg/mL. There was a study that suggested some flavonoids were obtained from the seeds of *O. indicum* collected from China; they mostly are in the flavone group and include chrysin, baicalein, baicalin, oroxylin A and their glucopyranosyl derivatives. Among them, baicalein exhibited the strongest *in vitro* antioxidant activity, which supported the results from this experiment [17]. Babu *et al.* [18] reported that chrysin, separated from the stem bark of this plant, promoted gastroprotective activity in both pylorus ligation-induced gastric lesions (PLU) and cold restrain-induced gastric ulcer (CRU) models. From the results, baicalein could be used as an active marker for the standardization and quality control of raw materials and extracts from the fruits of *O. indicum* in the future.

## 3. Materials and Methods

### 3.1. Preparation of Oroxylum Indicum Fruit Extracts

#### 3.1.1. Plant Material Preparation

The fruits of *Oroxylum indicum* were separately collected from Chiang Rai and Nakhon Pathom provinces, while the mature seeds were purchased from a traditional herbal shop in Bangkok in 2015. Plant samples were cleaned and dried in a hot air oven at 60 °C then ground using an electric mill (20 mesh sieve).

#### 3.1.2. Plant Extract Preparations

Each *O. indicum* fruit and seed powder was separately extracted by maceration using 95% ethanol and decoction with distilled water (plant:solvent ratio 1:20 *w*/*v*). Each extraction process was repeated three times. The extraction solutions were then combined, filtered and evaporated using a water bath to yield the dried extracts.

### 3.2. Determination of in Vitro Antibacterial Activity of *O. indicum Extracts Using* Disc Diffusion Assay 

The agar disc diffusion method was used to determine the diameter of the inhibition zone of each extract from the fruits and seeds of *O. indicum* [19]*.* The assays were performed against two clinically pathogenic bacteria (*Streptococcus suis* and *Staphylococcus intermedius*). Each extract was dissolved in 95% ethanol or sterile water at different concentrations (1.5625, 6.25, 25, 100, 125, 250, 500 and 1000 mg/mL). Then each solution was impregnated on to a small disc of filter paper and placed on the top of blood agar containing 100 µL of bacterial solution at a concentration of 1.5 × 10^8^ CFU/mL per plate (adjusted by comparing to 0.5 McFarland). The plate was incubated at 37 °C for 24 hours and the zone of inhibition was recorded. Amoxicillin/clavulanic acid 30 µg (AMC30), gentamicin 10 µg (CN10) and sulfamethoxazole-trimethoprim 25 µg (SXT25) were used as positive controls. All determinations were undertaken in triplicate and the average zone of inhibition was calculated with the standard deviation.

### 3.3. Determination of in Vitro Antioxidant Activity of O. indicum Extracts Using DPPH Scavenging Method 

The 2,2-diphenyl-1-picrylhydrazyl (DPPH) was dissolved in methanol to prepare the DPPH solution at a concentration of 207 µM. The DPPH solution (100 µL) was added to each plant extract solution with various concentrations ranging from 5–640 µg/mL in the same volume (100 µL). The mixture was mixed and kept in the dark for 15 min. The absorbance of each reaction solution was determined at a wavelength of 515 nm using a micro-plate reader. The percentage of inhibition for each reaction was then calculated, and EC_50_ values (µg/mL) were calculated from the linear equation from the curve between the percentage of inhibition and the solution’s concentration. Each experiment was conducted in triplicate. The EC_50_ value of each extract was expressed as the mean ± SD. The assays were performed as previously described [20].

### 3.4. Phytochemical Analysis

#### 3.4.1. Determination for Total Phenolic Content in *O. indicum* Extracts Using the Folin-Ciocalteu Method 

The Folin-ciocalteu assay was carried out according the method of Herald *et al.* [21] (2012) with gallic acid as the standard solution. A Folin-Ciocalteu reagent was added to each *O. indicum* fruit extract or standard gallic acid solution then the solution was kept in the dark at room temperature for 90 min. After that, 20% *w*/*v* sodium carbonate solution was added to each mixture. The absorbance of the reaction solution was determined at a wavelength of 765 nm using a micro-plate reader. The total phenolic compound was calculated from the standard curve of gallic acid and was expressed as g gallic acid equivalent per 100 g extract (g% GAE). Each experiment was carried out in triplicate. The average total phenolic content and standard deviation were then calculated.

#### 3.4.2. Determination of Total Flavonoid Content in *O. indicum* Extracts Using the Aluminium Chloride Method 

The aluminium chloride method was conducted using quercetin as the standard solution [22]. An aluminium chloride solution (10% *w*/*v*) was added to each plant extract or standard quercetin solution. The mixture was kept at room temperature for 30 min. The absorbance of the reaction solution was determined at a wavelength of 415 nm using a micro-plate reader. The total flavonoid content was calculated from the standard curve of quercetin and was expressed as g quercetin equivalent per 100 g extract (g% QE). Each experiment was carried out in triplicate. The average total flavonoid content and standard deviation were then calculated.

#### 3.4.3. Thin Layer Chromatography (TLC) Analysis

*Oroxylum indicum* extracts were spotted on a precoated silica gel 60 GF254 TLC plates. The plates were developed in two solvent systems, which were ethyl acetate:glacial acetic acid:formic acid:hexane (5:1:1:5, *v*/*v*/*v*/*v*) and ethyl acetate:toluene:formic acid (25:25:7.5, *v*/*v*/*v*). The developing distance was 85 mm. After being removed from the developing chamber, the TLC plates were air dried in a fume hood and examined under UV light at wavelengths of 254 and 366 nm and under UV light at a wavelength of 366 nm after spraying with a natural product/polyethylene glycol (NP/PEG) reagent. The TLC fingerprints of *O. indicum* fruit extracts were recorded.

### 3.5. Statistical Analysis

All data are reported as means ± standard deviation of the triplicate analyses. The least significant difference was used to compare the means (*p* < 0.05). Correlation coefficients R, to determine the relationship between two variables, were calculated using Pearson’s R test. All analyses were performed using SPSS for Windows, version16.0 (SPSS Inc., Chicago, IL, USA).

## 4. Conclusions

In the present study, the water and ethanol extracts from *Oroxylum indicum* fruits and seeds were tested for their *in vitro* antibacterial and antioxidant activities. The extracts from *O. indicum* fruits promoted moderate to intermediate antibacterial activities against both clinically isolated bacteria *Streptococcus suis* and *Staphylococcus intermedius*. The ethanol and water extracts from the seeds of *O. indicum* showed high *in vitro* antioxidant effects. Phytochemical study of the extracts showed a chromatographic band that could correspond to some phenolics and flavonoids including baicalein. This compound promotes a strong antioxidant effect, and could be used as the marker for the quality control of plant extracts and preparations from *O. indicum* in the future. Quantitative analysis of the active compound in *O. indicum* fruit and seed extracts should be further investigated in the future.

## Figures and Tables

**Table 1:** Antibacterial activity of *Oroxylum indicum* fruit and seed extracts against *Staphylococcus intermedius* and *Streptococcus suis* determined by the disc diffusion method.

Bacterial Strain	Concentration (mg/mL)	Zone of Inhibition (mm)								
		OPCD	OPCE	OPND	OPNE	OSBD	OSBE	AMC 30 µg	CN 10 µg	SXT 25 µg
*Staphylococcus intermedius*	1.5625	0	0	0	0	0	0	24.44 ± 0.73	15.00 ± 0.50	0
	6.25	0	0	0	0	0	6.78 ± 1.54			
	25	0	0	0	0	0	7.34 ± 1.87			
	100	0	8.33 ± 0.71 ^b^	0	8.56 ± 0.73 ^d^	7.83 ± 0.98	7.89 ± 4.89			
	125	0	9.28 ± 0.36 ^b^	0	10.22 ± 0.83	-	-			
	250	6.22 ± 0.44 ^a,1,2^	10.33 ± 0.43 ^b,1,3^	6.72 ± 0.51 ^c,3,4^	11.33 ± 0.83 ^2,4^	-	-			
	500	8.72 ± 0.44 ^a,5,6^	11.56 ± 0.46 ^b,5,7^	8.67 ± 0.50 ^c,7,8^	13.50 ± 1.32 ^5,6,8^	-	-			
	1000	11.17 ± 0.35 ^a,9^	12.67 ± 0.35 ^b^	11.17 ± 0.61 ^c,10^	15.11 ± 2.10 ^d,9,10^	-	-			
*Streptococcus suis*	1.5625	0	0	0	0	0	8.22 ± 0.97	32.56 ± 0.53	0	0
	6.25	0	0	0	0	0	8.56 ± 1.34			
	25	0	0	0	0	0	8.78 ± 1.86			
	100	0	0	0	0	0	7.56 ± 2.69			
	125	0	0	0	0	-	-			
	250	0	7.28 ± 0.44 ^f^	0	8.06 ± 0.39 ^h^	-	-			
	500	7.00 ± 1.00 ^e,11^	8.67 ± 1.00	7.72 ± 0.75 ^g,12^	11.33 ± 1.00 ^11,12^	-	-			
	1000	10.78 ± 0.67 ^e^	10.61 ± 0.99 ^f^	10.28 ± 0.91 ^g^	14.39 ± 2.47 ^h^	-	-			

OPCD = water extract from *O. indicum* fruits collected from Chiang Rai province, OPCE = ethanol extract from *O. indicum* fruits collected from Chiang Rai province, OPND = water extract from *O. indicum* fruits collected from Nakorn Pathom province, OPNE = ethanol extract from *O. indicum* fruits collected from Nakorn Pathom province, OSBD = water extract from *O. indicum* seeds purchased from Bangkok, OSBE = ethanol extract from *O. indicum* seeds purchased from Bangkok, AMC 30 µg = Amoxicillin/clavulanic acid 30 µg, CN 10 µg = gentamicin 10 µg and SXT 25 µg sulfamethoxazole-trimethoprim 25 µg. Means followed by the same letter (a, b, c, d, e, f, g, h) and the same number (1, 2, 3, 4, 5, 6, 7, 8, 9, 10, 11, 12) are significantly different (*p* < 0.05).

**Table 2:** Antioxidant activity, total phenolic and total flavonoid contents of *O. indicum* extracts.

Extracts	DPPH Assay EC_50_ (µg/mL)	Total Phenolic Content (g% GAE)	Total Flavonoid Content (g% QE)
OPCD	171.30 ± 8.46 ^e^	3.22 ± 0.10 ^b^	1.68 ± 0.13 ^a^
OPCE	65.89 ± 5.48 ^c^	4.57 ± 0.45 ^c^	4.68 ± 0.12 ^c^
OPND	220.50 ± 14.50 ^f^	2.70 ± 0.05 ^a^	1.49 ± 0.18 ^a^
OPNE	84.29 ± 5.16 ^d^	4.39 ± 0.19 ^c^	3.76 ± 0.67 ^b,c^
OSBD	38.87 ± 3.90 ^b^	8.21 ± 0.62 ^d^	3.94 ± 0.22 ^b^
OSBE	26.33 ± 0.84 ^a^	10.66 ± 0.27 ^e^	7.16 ± 0.06 ^d^
Ascorbic acid	3.86 ± 0.12	-	-
Baicalein	3.17 ± 0.05	-	-

OPCD = water extract from *O. indicum* fruits collected from Chiang Rai province, OPCE = ethanol extract from *O. indicum* fruits collected from Chiang Rai province, OPND = water extract from *O. indicum* fruits collected from Nakorn Pathom province, OPNE = ethanol extract from *O. indicum* fruits collected from Nakorn Pathom province, OSBD = water extract from *O. indicum* seeds purchased from Bangkok, OSBE = ethanol extract from *O. indicum* seeds purchased from Bangkok. Different superscript letters (a, b, c, d, e) in the same column show significant difference (*p* < 0.05).
